# The Power of Collaboration in Facilitating Mobile Technology Adoption in Health Care

**DOI:** 10.2196/60350

**Published:** 2024-07-08

**Authors:** Weijing Fan, Guobin Liu

**Affiliations:** 1 Department of Peripheral Vascular Surgery, Institute of Surgery of Traditional Chinese Medicine Shuguang Hospital Affiliated to Shanghai University of Traditional Chinese Medicine Shanghai China

**Keywords:** social workers, government, mobile technology, mobile apps, fitness trackers, SMS, SMS text messaging, physical activity, exercise, sedentary behavior, Middle East, Africa, movement, physical inactivity, smartphone, mobile phone, mobile health, mHealth, digital health, behavior change, intervention

We read the recently published article titled “The Use of Mobile Technologies to Promote Physical Activity and Reduce Sedentary Behaviors in the Middle East and North Africa Region: Systematic Review and Meta-Analysis” by Tong HL et al [1] with great interest. The authors explored the impact of mobile technologies on physical activity and sedentary habits in the Middle East and North Africa (MENA) region. Even though solid conclusions were not obtained due to insufficient number and quality of the included studies, the analysis yielded some promising insights. However, several aspects merit further exploration.

Primarily, the authors did not search the commonly used databases PubMed, Web of Science, and Cochrane in their systematic review [2]. An explanation for how to ensure completeness of the search is thus needed. Second, behavior change is usually a gradual process of change, so the frequency and duration of the intervention have an important impact on the outcome of behavior change; too much repetitive information can lead to user fatigue and ignored messages, and the authors did not analyze the relevant information. Third, the authors should conduct a more specific analysis of potential confounding factors that may affect the outcome, as well as variables such as the nature of work, family support, and education that significantly affect the actual effect of mobile technologies [3]. Without controlling for these variables, attributing their effects to mobile technologies will be very challenging.

Finally, the authors emphasize the crucial role that governments play in the context of mobile technologies—a point well-taken. Social workers also play a key part in this process. Many countries in the MENA region have vast differences in terms of economy, politics, and culture [4]; these countries can leverage their understanding of local economics, culture, and health determinants to advocate for policies that boost the uptake of mobile technologies uptake. Additionally, social workers can foster dialogue between medical professionals and government officials, offering social insights to enhance the deployment of mobile technologies. We look forward to seeing governments, social workers, doctors, and families collaborate to leverage mobile technologies for improved public health management.

## Abbreviations

MENA: Middle East and North Africa
